# A Cuproptosis-Related Gene Model For Predicting the Prognosis of Clear Cell Renal Cell Carcinoma

**DOI:** 10.3389/fgene.2022.905518

**Published:** 2022-08-11

**Authors:** Wangli Mei, Xiang Liu, Xuyang Jia, Liang Jin, Shiyong Xin, Xianchao Sun, Jiaxin Zhang, Bihui Zhang, Yilai Chen, Jianping Che, Weiguo Ma, Lin Ye

**Affiliations:** ^1^ Department of Urology, Shanghai East Hospital, School of Medicine, Tongji University, Shanghai, China; ^2^ Department of Urology, Shanghai Tenth People’s Hospital, School of Medicine, Tongji University, Shanghai, China; ^3^ Department of Urology, Tongxin People’s Hospital, Ningxia, China; ^4^ Department of Metabolic Surgery, Shanghai Tenth People’s Hospital, School of Medicine, Tongji University, Shanghai, China; ^5^ Department of Urology, Karamay People’s Hospital, Xinjiang, China; ^6^ Department of Urology, Shanghai Putuo District People’s Hospital, School of Medicine, Tongji University, Shanghai, China

**Keywords:** cuproptosis, clear cell renal cell carcinoma (ccRCC), prognostic signature, immune infiltration, bioinformatics analysis

## Abstract

Despite advances in its treatment, patients diagnosed with clear cell renal cell carcinoma (ccRCC) have a poor prognosis. The mechanism of cuproptosis has been found to differ from other mechanisms that regulate cell death, including apoptosis, iron poisoning, pyrophosphate poisoning, and necrosis. Cuproptosis is an essential component in the regulation of a wide variety of biological processes, such as cell wall remodeling and oxidative stress responses. However, cuproptosis-related genes’ expression in ccRCC patients and their association with the patient’s prognosis remain ambiguous. Evaluation of The Cancer Genome Atlas (TCGA) identified 11 genes associated with cuproptosis that were differently expressed in ccRCC and nearby nontumor tissue. To construct a multigene prognostic model, the prognostic value of 11 genes was assessed and quantified. A signature was constructed by least absolute shrinkage and selection operator (LASSO) Cox regression analysis, and this signature was used to separate ccRCC patients into different risk clusters, with low-risk patients having a much better prognosis. This five-gene signature, when combined with patients’ clinical characteristics, might serve as one independent predictor of overall survival (OS) in ccRCC patients. Gene ontology (GO) and Kyoto Encyclopedia of Genes and Genomes (KEGG) analysis demonstrated that cuproptosis-related genes were enriched in patients with ccRCC. Then, quantitative real-time PCR (qPCR) was employed to verify these genes’ expression. Generally, research has indicated that cuproptosis-related genes are important in tumor immunity and can predict OS of ccRCC patients.

## Introduction

Renal cell carcinoma (RCC) is the most common type of kidney cancer, accounting for 2.2% of the global cancer burden and 1.8% of tumor-related deaths (Sung et al., 2021). CcRCC is the most frequent pathogenic subtype in RCC, being present in about 80% of patients with RCC (Gulati and Vaishampayan, 2020). Over the past few decades, there have been increases in both the incidence of ccRCC and deaths from this disease (Ljungberg et al., 2011). The prognosis for people with ccRCC remains dismal, despite recent breakthroughs in diagnosis and treatment, particularly for those with advanced-stage tumors and tumors that have metastasized to other organs (Dutcher, 2013). Because early diagnosis is critical for effective treatment, new diagnostic markers and prognostic signatures are urgently needed.

The process of cuproptosis, which is thought to have some key roles in the treatment of malignancies, is presented in the early stages of development (Steinbrueck et al., 2020). Copper ion is a cofactor that is critical for the growth and progression of all organisms, being involved in many biological processes, including photosynthesis and respiration (Culotta et al., 1999), cell wall remodeling, and oxidative stress responses (Yruela, 2009). Intracellular copper concentrations are maintained at extremely low levels by an active homeostatic mechanism that uses trans-concentration gradients to avoid the accumulation of free copper within cells (Tanzi et al., 1993). Even low intracellular levels of copper can poison cells, resulting in cell death (Bondarczuk and Piotrowska-Seget, 2013). Toxic copper ion carriers and copper chelating agents are regarded as anticancer medicines because of their ability to inhibit the growth of malignancies (Wang and Franz, 2016). In addition to acting as small chaperones, copper ionophores enhance cell death by increasing the intracellular accumulation of copper (Tsvetkov et al., 2022). Alterations in the ability of copper to form bonds with small molecules reduce the ability of copper to induce cell death (Tsvetkov et al., 2019).

The mechanism that cuproptosis induces cell death has been shown to differ from other mechanisms that control cell death, including apoptosis, iron poisoning, pyrophosphate poisoning, and necrosis (Tsvetkov et al., 2022). The roles of cuproptosis-related genes in the development and outcome of ccRCC have not yet been determined. It is unclear whether the cuproptosis-related gene signature has a significant effect on the prognosis of ccRCC patients and their immune microenvironment. The present study evaluated genes associated with cuproptosis to examine if a cuproptosis-related gene signature predicts OS of ccRCC patients and modulate the immune microenvironment.

## Materials and Methods

### Data Collection

RNA-seq data for ccRCC were acquired from TCGA (Tomczak et al., 2015), including 72 normal kidney samples and 539 ccRCC samples. Results were reported as fragments per kilobase per million. “Limma” package supplied a scale approach for normalizing gene expression data (Ritchie et al., 2015). Clinical data, including race, sex, and tumor stage, were also obtained for 530 samples in TCGA. Data of 91 additional samples were downloaded on International Cancer Genome Consortium (ICGC) website, in which most of the data were obtained from a European population. Thirteen cuproptosis-related genes had been identified in previous investigations and were included in this study (Tsvetkov et al., 2022). Supplementary Appendix Table S1 shows visual representations of these genes.

### Construction and Validation of the Signature

Differentially expressed genes (DEGs) between tumor and para-carcinoma tissues in TCGA were determined by “limma” package, with cutoffs that included p and false discovery rate (FDR) < 0.05. Relationships between the expression of 13 cuproptosis-related genes and patient survival in TCGA were determined by univariate Cox analysis. *p* < 0.05 was chosen as a threshold to maintain the predictive performance of this signature. Further research was conducted on overlapped prognostic DEGs. Interacting networks were established using Search Tool for the Retrieval of Interacting Genes (STRING, version 11.5(Jensen et al., 2009). Target genes were further identified using lasso Cox regression analysis (Friedman et al., 2010), and a prognostic model was determined using “glmnet” package. The prognostic significance of genes in this signature was also assessed by multivariate Cox analysis. Genes and coefficients were gathered, and the penalty parameter (λ) was computed using the smallest possible criterion. Total risk scores were based on each gene’s normalized expression and were expressed using the algorithm: risk score = $\sum_{i}^{\text{n}}Xi\text{*}Yi$ ( X : coefficients, Y : gene expression). The median risk score was calculated, with patients having risk scores above and below the median assigned to subgroups, respectively. The ability to separate samples into two clusters was assessed by principal component analysis (PCA) of this signature using the “stats” R package. OS of ccRCC patients indifferent risk clusters were compared by Kaplan–Meier (KM) analysis by the “survminer” package, and 1-, 3-, and 5-years OS was assessed by receiving operating characteristic (ROC) curve analysis using “survival”, “survminer”, and “timeROC” packages to assess the predictive potential of this signature.

### Independent Prognostic Analysis of This Signature

Data on patient age, gender, race, tumor stage, survival time, and survival status were obtained for 611 samples in TCGA and 91 samples in ICGC-RECA-EU. The association between these factors and expression of cuproptosis-related genes was estimated by Cox regression analyses, with outcomes visualized by forest maps. Multivariate Cox analysis was also employed by “rsm” package (Lenth, 2009), with the resulting nomogram and calibration curve employed to determine whether this nomogram was accurate.

### Validation of Genes in This Signature

Data on the expression of each cuproptosis-related gene in normal and ccRCC tissue samples, as assessed by immunohistochemistry, were obtained from Human Protein Atlas (Thul and Lindskog, 2018), and employed to validate genes’ protein expression. Additionally, KM curves based on genes’ expression levels were utilized to evaluate the association between each gene and OS in these ccRCC patients.

### Functional Enrichment Analysis and Immunocorrelation Analysis

Differential analyses comparing groups with differing levels of risk were assessed by “limma” package, with criteria for difference being |log2FC| ≥ 1 and FDR <0.05. Immune scores in patients with ccRCC were obtained by Estimation of Stromal and Immune cells in Malignant Tumor tissues using Expression (ESTIMATE) (Yoshihara et al., 2013), with groups having high and low tumor ESTIMATE scores subjected to differential analyses using as criteria |log2FC| ≥ 1 and FDR <0.05. Similar degrees in the two sets were evaluated by Venn diagrams. Finally, these genes were used to analyze gene functions and pathways connected to the risk and immunological scores. Identified DEGs were subjected to KEGG, Disease Ontology (DO), and GO analyses, including analyses of Biological Process (BP), Cellular Component (CC), and Molecular Function (MF), using “clusterProfiler” package (Wu et al., 2021). Infiltration scores of immune cells and pathways (Supplementary Appendix Table S3) were analyzed using a heatmap. Linkages between immune cells and pathways were determined by correlation analysis. Infiltration scores were investigated by Gene Set Variation Analysis (GSVA) (Hanzelmann et al., 2013) using the “GSVA” package with single-sample gene set enrichment analysis (ssGSEA, https://software.broadinstitute.org/cancer/software/genepattern/). All overlapping genes were subjected to univariate Cox regression analysis to find prognostic genes with *p* < 0.05. The correlations of these genes with immune cells and pathways were assessed using the “corr.test” function in the “psych” package (https://cran.r-project.org/package=psych).

### Cell Culture

CcRCC cell line 786-O and human proximal tubular epithelial cell line HK-2 were cultured in Dulbecco’s modified Eagles’ medium (DMEM; Gibco, Grand Island, NY, United States) supplemented with 10% fetal bovine serum (FBS; Hyclone, Logan, UT, United States). Cultivation conditions were maintained with 37°C and 5% CO_2_.

### Quantitative Real-Time PCR

Total RNA was extracted from 786-O and HK-2 cell lines, and reverse-transcribed into cDNA. The expression of five genes in our signature was quantified with qPCR. Primers of genes were as follows: *ATP7B*, 5′-TCT​GTG​CTG​ATT​GGA​AAC​CGT​GAG-3′ (forward) and 5′-CAC​CGT​CAA​TAG​CCA​CCA​GGA​TG-3′ (reverse); *DBT*, 5′-TTC​TCT​GAA​CCC​ACC​TCT​GTC​CTG-3’ (forward) and 5′-ACC​TGG​TCT​GTC​TGA​CTG​TCT​GAG-3’ (reverse); *DLAT*, 5′-TTG​ATG​TCA​GTG​TTG​CGG​TCA​GTA​C-3’ (forward) and 5′-GTG​GCT​GTA​GTT​TAC​CCT​CTC​TTG​C-3’ (reverse); *PDHB*, 5′-CGA​GGG​CTG​TGG​AAG​AAA​TAT​GGA​G-3’ (forward) and 5′-GGC​TTG​CAT​GGA​GAA​ATT​GAA​GGT​C-3’ (reverse); *LIAS*, 5′-AAT​AAC​AGA​GGT​GGT​GCC​AGA​ATG​C-3’ (forward) and 5′-GTG​CTG​GGA​TTA​CAG​GCG​TGA​G-3’ (reverse). The levels of mRNA expression were estimated using the 2^−Δ^CT method after being normalized to β-actin expression levels.

### Statistical Analyses

The ability of this model to predict OS was evaluated in TCGA and ICGC clinical cohorts. OS of patients in subgroups was evaluated by K-M curves and compared by log-rank tests. This independent prognostic significance of this signature was evaluated by Cox regression analyses. Statistical analyses were employed by R software (version 4.41).

## Results

### Differential Analyses of Tumor and Normal Tissues

**FIGURE 1:**
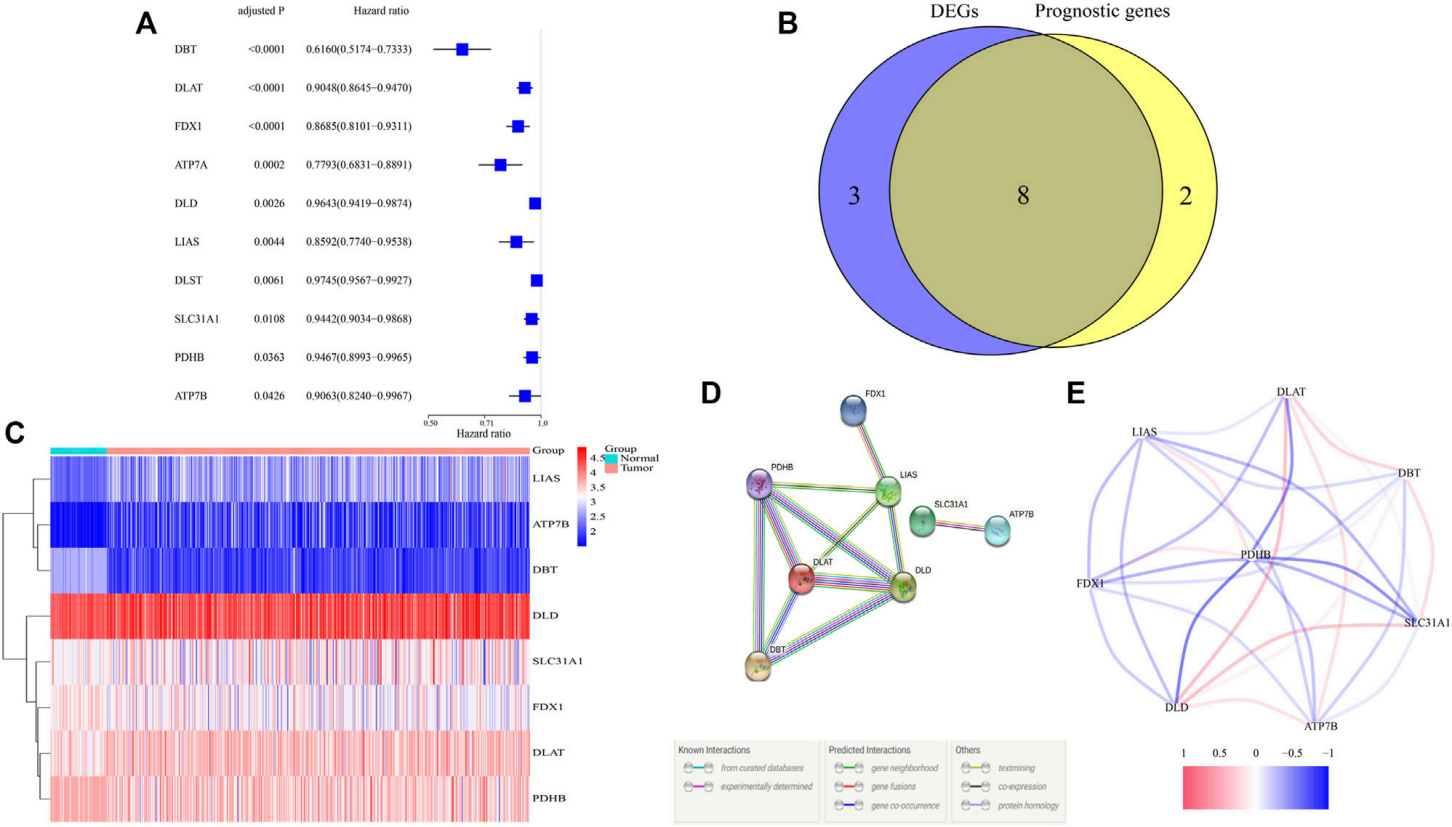
Identification of candidate cuproptosis-related genes in TCGA. **(A)** Univariate Cox regression analysis of 10 cuproptosis-related genes with *p* < 0.05 in patients with ccRCC. **(B)** Venn diagram identifying differentially expressed genes correlated with OS. **(C)** Heatmap of the expressions of eight overlapping genes. **(D,E)** PPI (interaction score = 0.4) and correlation network of cuproptosis-related genes.

Differential analysis of expression of 13 cuproptosis-related genes between tumor and adjacent nontumor tissue samples in TCGA identified 11 DEGs with p and FDR <0.05 (Supplementary Appendix Table S1). Evaluation of the prognostic significance of 13 genes by univariate Cox analysis showed that 10 genes had p and FDR <0.05 (Figure 1A, Supplementary Appendix Table S1). A Venn diagram found an overlap of eight of these DEGs that were associated with prognosis (Figure 1B). A heatmap showed that the levels of expression of *ATP7B*, *DLAT*, *DLD*, *LIAS,* and *SLC31A1* were upregulated and the levels of expression of *DBT*, *FDX1,* and *PDHB* downregulated in tumor samples (Figure 1C). *DLAT*, *DLD*, *DBT*, *PDHB*, and *LIAS* have been identified as hub genes in the interaction network, with a score ≥0.4 needed for each interaction (Figure 1D). The eight identified genes were found to form a strong association network (Figure 1E).

### Cuproptosis-Related Prognostic Model in TCGA

**FIGURE 2:**
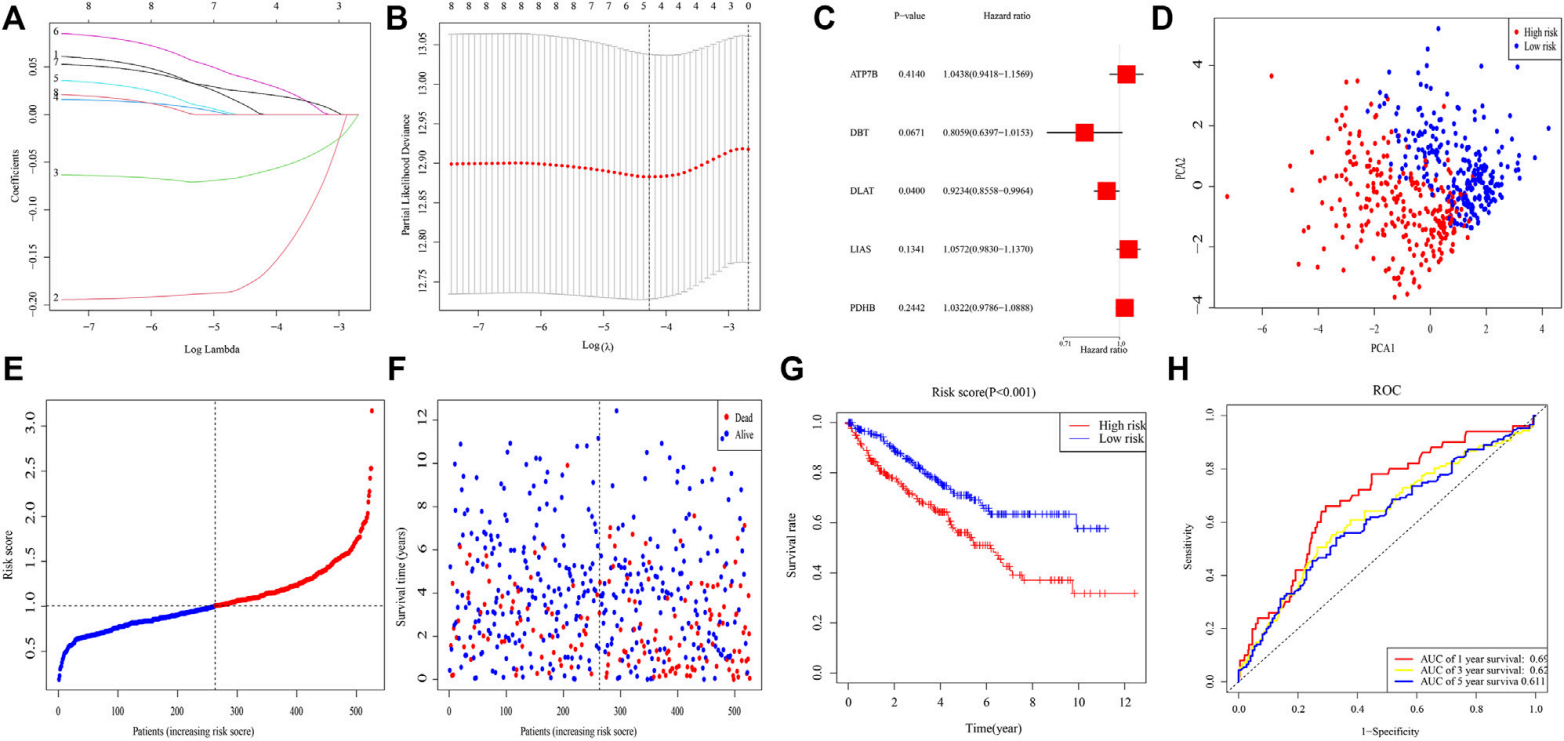
Risk signature in TCGA. **(A)** Lasso regression analysis of eight genes. **(B)** Cross-validation of results of lasso regression analysis. **(C)** Multivariable Cox regression analysis of genes determined by lasso regression analysis. **(D)** PCA plot of patients with ccRCC. **(E)** Distribution of ccRCC patients based on risk scores. **(F)** Distributions of OS status, OS, and risk scores. **(G)** KM curves for OS of ccRCC patients in different clusters. **(H)** ROC curves of this signature.

After excluding samples with missing data on survival, 526 samples were investigated. LASSO Cox regression analysis of eight genes illustrated that a five-gene signature provided the optimal value (Figures 2A,B). Multivariable Cox analysis of these five genes yielded *p*-values for the *ATP7B*, *DBT*, *DLAT*, *LIAS*, and *PDHB* genes of 0.414, 0.067, 0.040, 0.134, and 0.244, respectively (Figure 2C). Risk score was calculated as (0.043* *ATP7B* exp.) + (−0.216* *DBT* exp.) + (−0.080* *DLAT* exp.) + (0.056* *LIAS* exp.) + (0.032* *PDHB* exp.). A median score was utilized to separate 526 patients into different risk groups of 263 patients each, with PCA showing that these patients were clearly separated into two distinct subsets (Figure 2D). The death rate was higher and time to death was shorter in high-risk clusters (Figures 2E,F), with K-M analysis showing that OS differed significantly in two clusters (*p* < 0.001) (Figure 2G). ROC analysis showed that areas under the curve (AUCs) for 1-, 3-, and 5-year survival were 0.692, 0.624, and 0.611, respectively, (Figure 2H).

### External Validation of Our Signature

**FIGURE 3:**
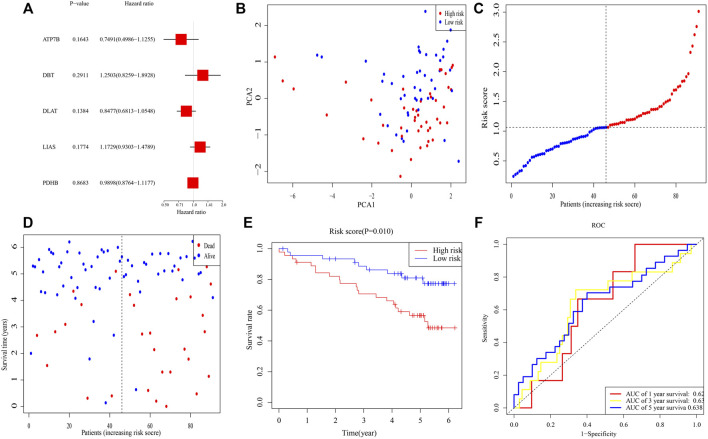
Validation of the risk signature in ICGC. **(A)** Multivariable Cox regression analysis of the five genes determined by lasso regression analysis. **(B)** PCA plot of patients with ccRCC. **(C)** Distribution of ccRCC patients based on the signature. **(D)** Distributions of OS status, OS, and risk scores. **(E)** K-M curves for OS of ccRCC patients in risk subgroups. **(F)** AUC of time-dependent ROC curves.

To validate this signature, RNA-seq and clinical data from 91 individuals with ccRCC were retrieved from the ICGC (RECA-EU) cohort. Multivariable Cox analysis of these five genes yielded *p*-values for the *ATP7B*, *DBT*, *DLAT*, *LIAS*, and *PDHB* genes of 0.164, 0.291, 0.138, 0.177, and 0.868, respectively, (Figure 3A). Median risk score was calculated for these patients, and 91 patients were separated into 45 high- and 46 low-risk patients. PCA demonstrated that these samples were clearly divided into two subgroups (Figure 3B). As in the TCGA cohort, the death rate was higher and the time to death shorter in high-risk clusters (Figures 3C,D). K-M analysis demonstrated that OS differed significantly in subgroups (*p* = 0.010) (Figure 3E). Moreover, ROC analysis illustrated that AUCs for 1-, 3-, and 5-year survival were 0.62, 0.63, and 0.63 (Figure 3F).

### Independent Prognostic Value of This Signature

**FIGURE 4:**
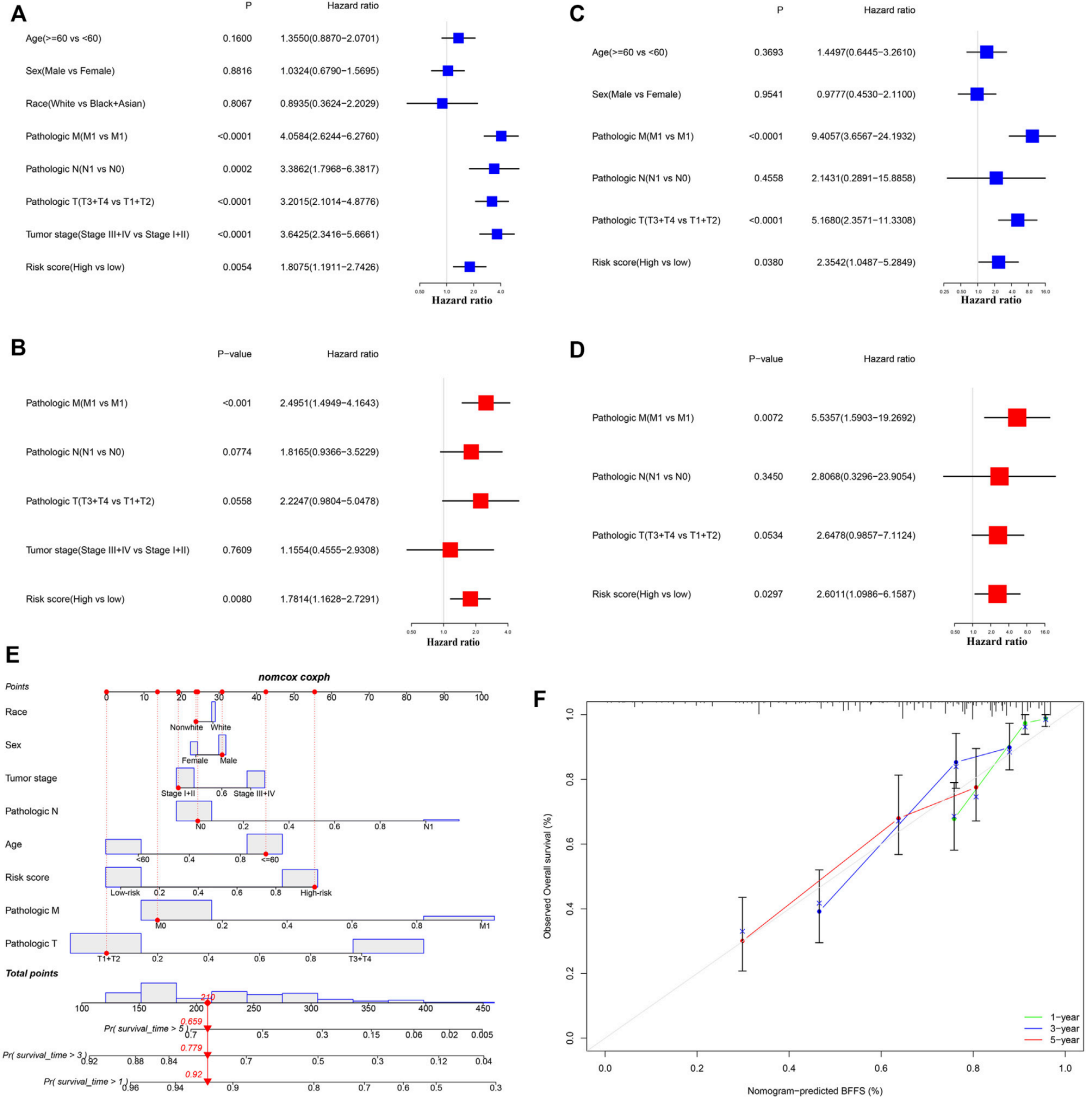
Cox regression analyses. **(A,B)** Univariate and multivariate Cox regression analyses of patients in TCGA. **(C,D)** Univariate and multivariate Cox regression analyses in ICGC. **(E)** Nomogram-based gene signatures for prediction of 1-, 3- and 5-year OS. **(F)** Calibration plots for agreement tests between predicted and actual OS.

After excluding samples with incomplete clinical data, 245 patients from TCGA and 80 from ICGC were analyzed further. Table 1 illustrates the clinical information of these individuals. Cox regression analysis was employed to assess if this signature had independent predictive significance. Univariate Cox regression analyses declared that risk score was a significant predictor of OS in patients with ccRCC, both in TCGA [*p* = 0.005, hazard ratio (HR) = 1.806, 95% confidence interval (CI): 1.191–2.743] and ICGC (*p* = 0.038, HR = 2.354, 95% CI: 1.049–5.285) (Figures 4A,B). Multivariable Cox regression analyses also showed that our signature was an independent predictor of OS in patients with ccRCC, both in TCGA (*p* = 0.008, HR = 1.781, 95% CI: 1.163–2.729) and ICGC (*p* = 0.030, HR = 2.601, 95% CI: 1.099–6.159) (Figures 4C,D). A predictive nomogram for OS was formulated using all relevant clinical variables for patients in TCGA (Figure 4E). The red dot represents the clinical feature score of a patient from TCGA with 210 points, indicating that this patient had probabilities of survival at 1, 3, and 5 years of 92.0, 77.9, and 65.9%, respectively. Calibration curves were also determined for 1-, 3-, and 5-year OS of ccRCC patients (Figure 4F).

**TABLE 1:** Baseline characteristics of the patients in different risk groups in TCGA and ICGC cohorts.

Clinical characteristic (samples)		TCGA		ICGC	
		Low risk	High risk	Low risk	High risk
Age (years)	<60	51	53	19	15
	≥60	79	62	23	23
Sex	Female	75	40	23	15
	Male	73	75	19	23
Race	White	122	105		
	Non-white	8	10		
Pathologic M	M0	26	89	39	34
	M1	15	26	3	4
Pathologic N	N0	122	108	41	37
	N1	8	7	1	1
Pathologic T	T1 + T2	80	64	35	26
	T3 + T4	50	51	7	12
Tumor stage	Stage I + II	77	55		
	Stage III + IV	53	60		

### Validation of Each Gene in This Signature

**FIGURE 5:**
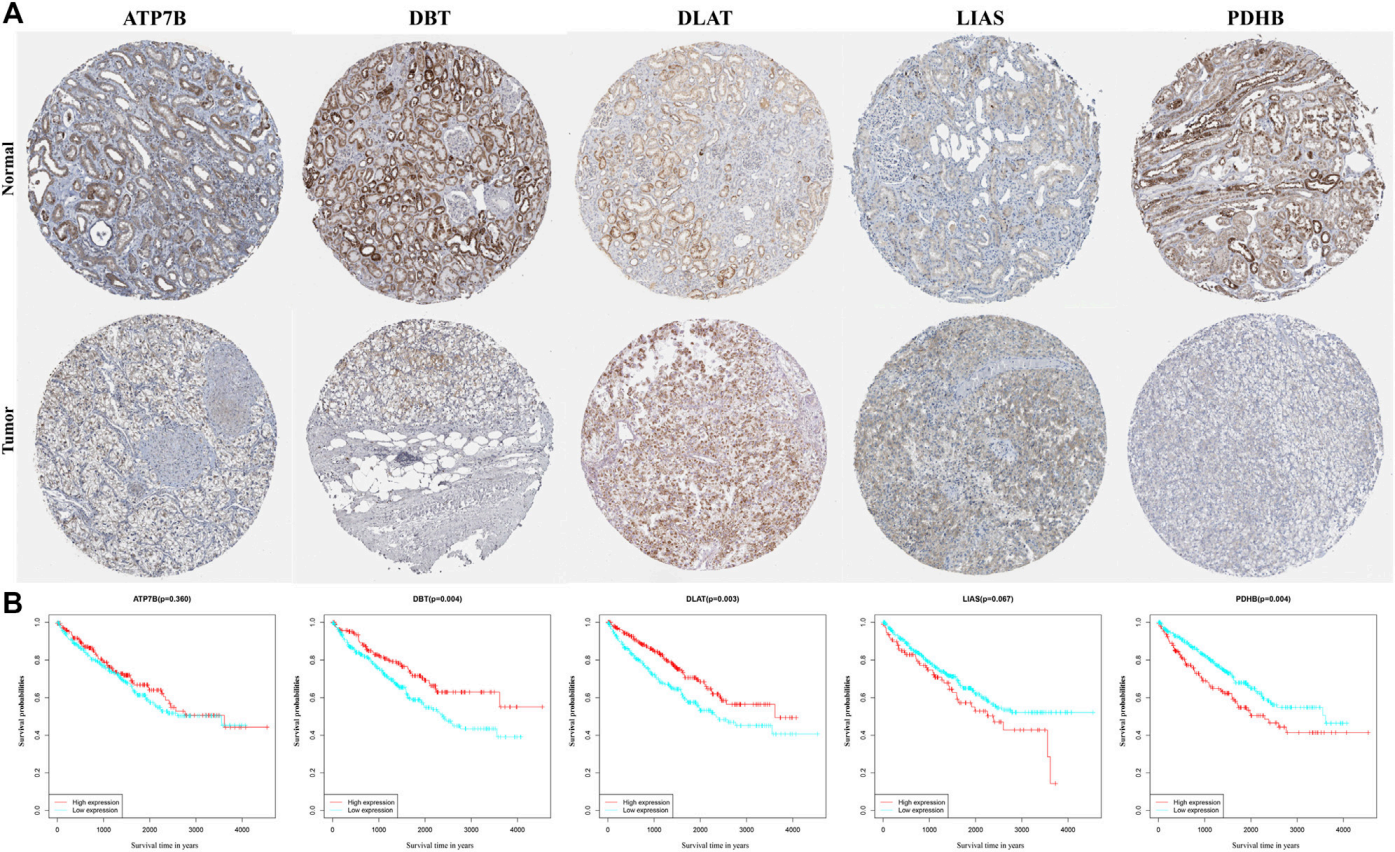
Validation of each gene in the gene signature. **(A)** Levels of expression of genes, as determined immunohistochemically, in samples from the Human Protein Atlas database. **(B)** KM curves for OS of ccRCC patients based on the expression of genes.

Data on the levels of expression of the proteins encoded by the five genes, as determined immunohistochemically, were retrieved from Human Protein Atlas database. Levels of expression of the *ATP7B*, *DLAT*, and *LIAS* proteins were higher, whereas the levels of expression of *DBT* and *PDHB* were lower, in tumor samples than in non-tumorous kidney tissue, in accordance with the results of differential mRNA analysis (Figure 5A). K-M analysis of OS in patients separated into two clusters based on the median expression of proteins (Supplementary Appendix Table S2) showed statistically significant correlations between the levels of expression of *DBT* (*p* = 0.004), *DLAT* (*p* = 0.003), and *PDHB* (*p* = 0.004) and OS in these patients (Figure 5B).

**FIGURE 6:**
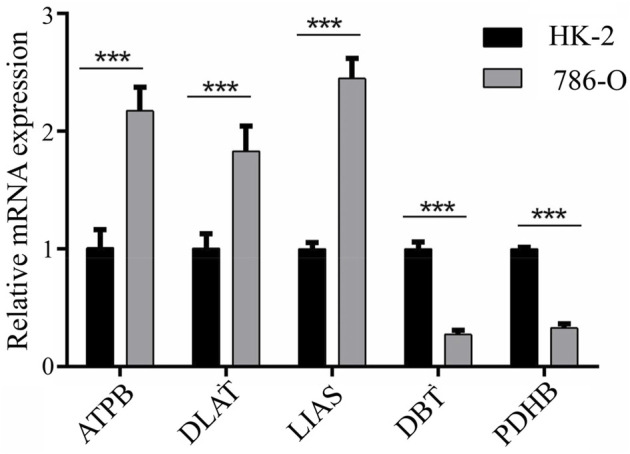
Results of qPCR for five genes between HK-2 and 786-O cell lines.

Expression of *ATP7B*, *DBT*, *DLAT*, *LIAS*, and *PDHB* was assessed with qPCR in 786-O and HK-2 cell lines. The results demonstrated that *ATP7B*, *DLAT,* and *LIAS* were upregulated in the 786-O cell line, which represented human renal clear cell adenocarcinoma cell, compared to the HK-2 cell line; while *DBT* and *PDHB* were found to be downregulated in 786-O cells (Figure 6). The experiment verified the aforementioned conclusion.

### Functional Enrichment and Immune Activity Analysis

**FIGURE 7:**
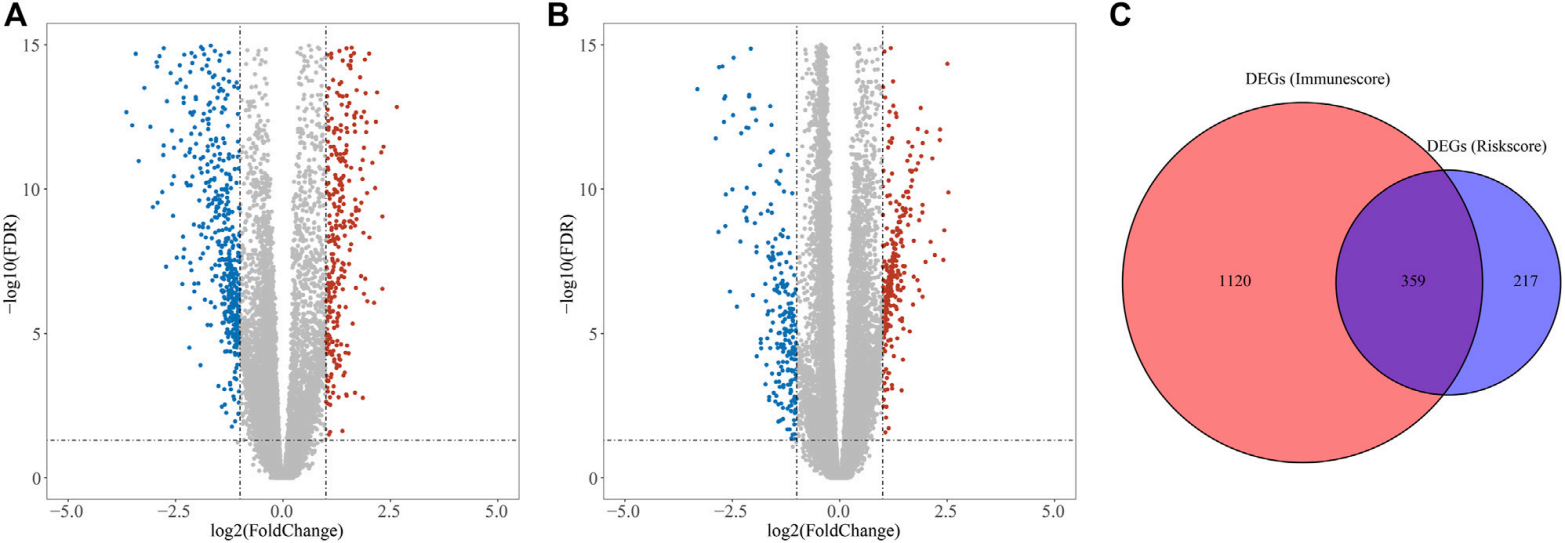
DEGs for functional enrichment and immune activity analysis. **(A)** Heatmap of DEGs in the high- and low-risk groups. **(B)** Heatmap of DEGs between high and low levels of immune cell infiltration groups. **(C)** Overlapping genes of these DEGs.

**FIGURE 8:**
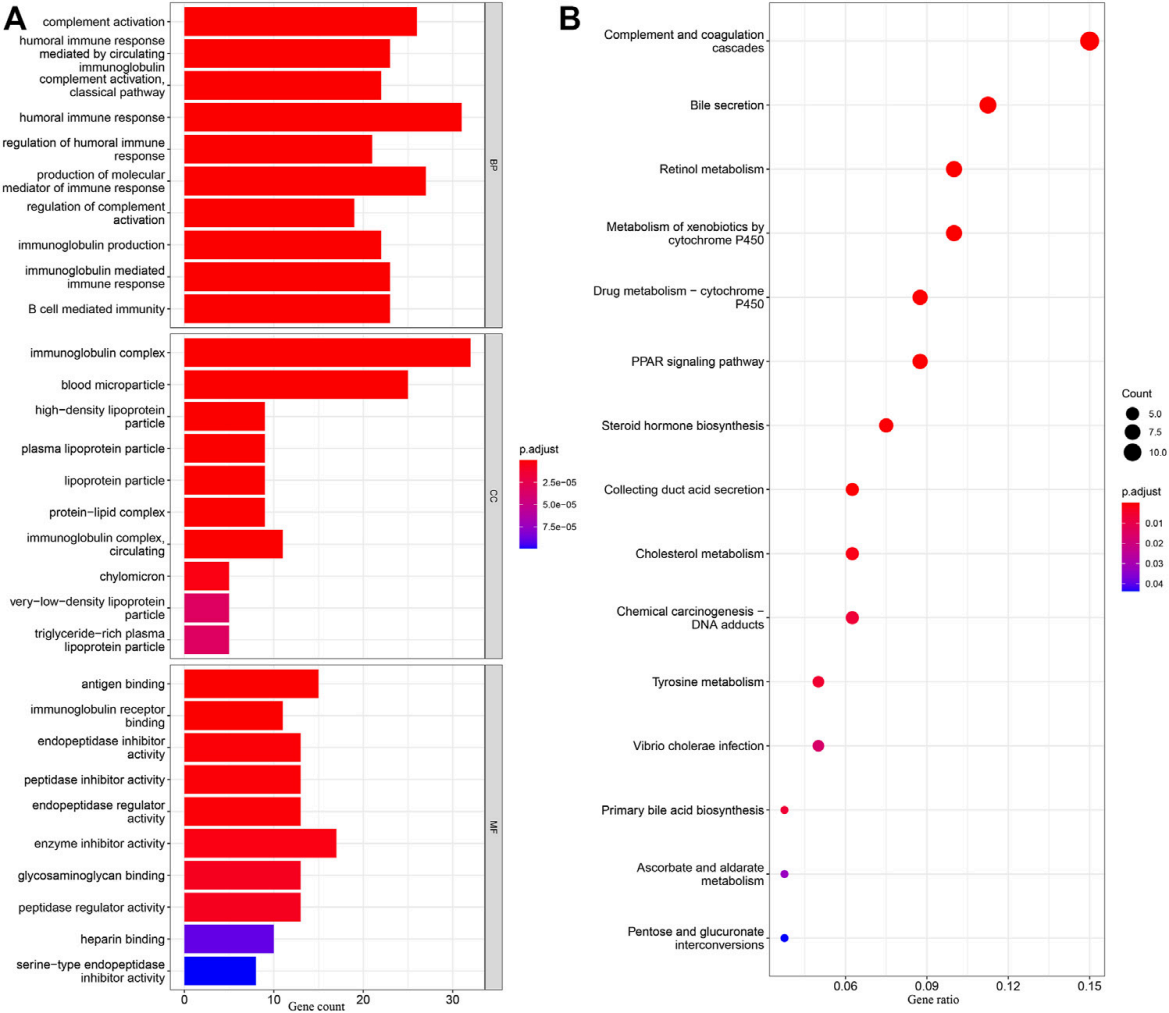
KEGG and GO analyses in TCGA. **(A)** Barplot graph for GO enrichment, with bar length representing the degree of enrichment and color representing the degree of difference. **(B)** Bubble graph for KEGG enrichment, with larger bubbles representing more genes enriched and increasing depth of red representing an increasing degree in difference.

DEGs in different risk subgroups were assessed to seek potential biological activities and pathways associated with this signature for ccRCC. Assessment of the TCGA cohort identified 576 DEGs that met the criteria of FDR < 0.05 and |log2FC | ≥ 1 (Figure 7A). Using the same criteria, 1479 DEGs were identified in comparisons of patients with different levels of immune cell infiltration (Figure 7B). A comparison of these two sets of DEGs identified 359 overlapping DEGs (Figure 7C). GO enrichment and KEGG analyses illustrated that these genes were involved in immunoglobulin complex formation, humoral immune responses, regulation of the immune effector process, and complement and coagulation cascades (Figures 8A,B). Outcomes of the DO analysis have also been included in the Supplementary Materials.

**FIGURE 9:**
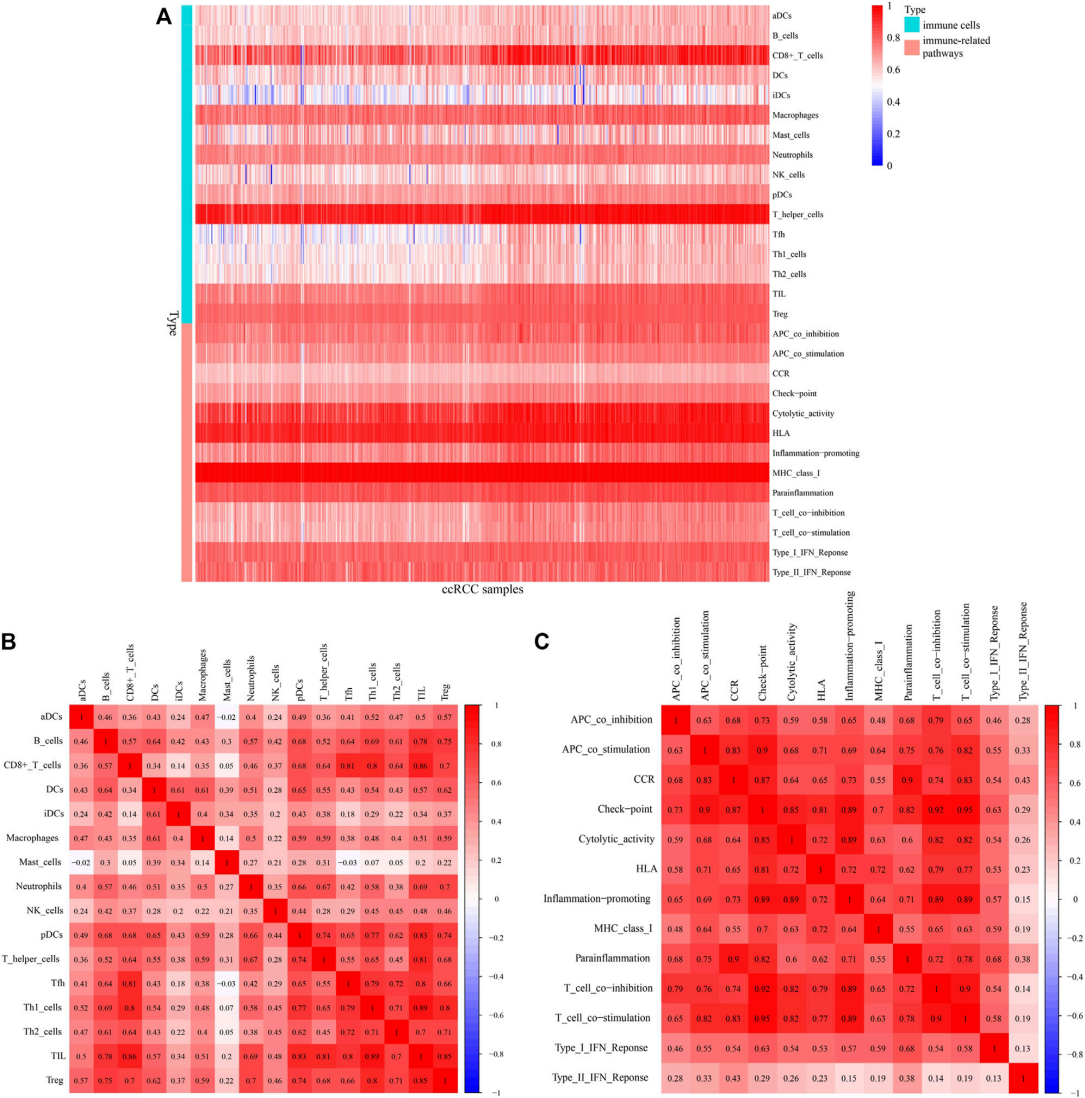
Immunocorrelation analysis. **(A)** Heatmap of infiltration scores of immune cells and pathways. **(B,C)** Correlations between different immune cells and pathways, with redder color indicating a higher degree of correlation.

A heatmap shows infiltration scores of 16 immune cells and activation of 13 immune pathways (Figure 9A). CD8^+^ T cells, T helper cells, cytolytic activity, and human leukocyte antigen (HLA) were significantly associated with ccRCC patients. Figures 9B,C shows the associations between ccRCC infiltrating immune subsets and various immune cells, as well as several immune-related pathways, representing the interactions and degree of activity of these immune cells and pathways, with correlation coefficients closer to one indicating greater associations between immune cells and immunological pathways.

**FIGURE 10:**
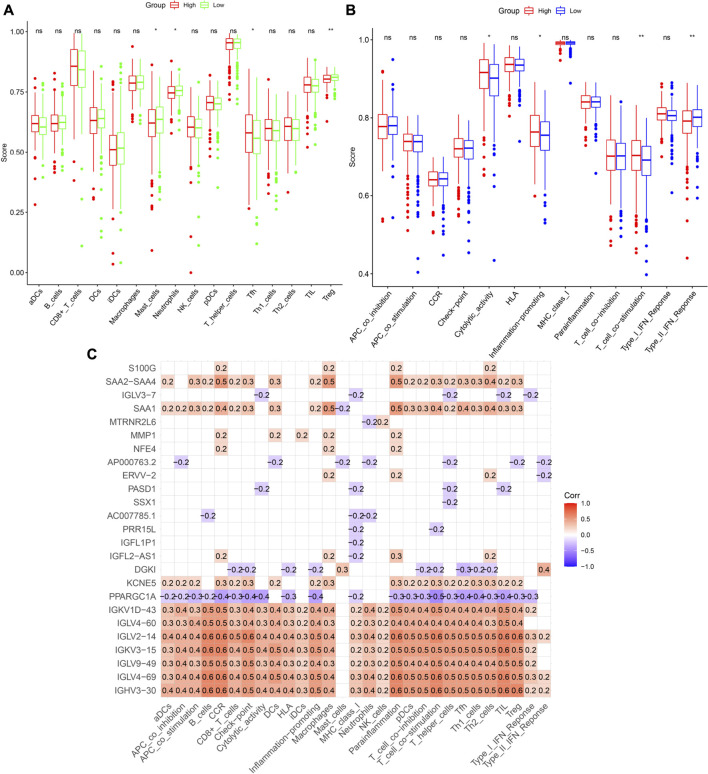
Immune infiltration analysis. **(A,B)** Comparison of enrichment scores of immune cells and pathways. **(C)** Correlation between prognosis-related DEGs and immune cells or pathways. The number in the grid indicates the correlation coefficient. Red represents positively correlated, while violet represents negatively correlated.

The connection between patient risk scores and immune state, including the infiltration scores of 16 types of immune cells and the activity of 13 pathways, was assessed by ssGSEA. Levels of infiltration of immune cells, such as mast cells, neutrophils, and Treg cells, were lower, whereas the level of T follicular helper (Tfh) cells was higher, in the high-risk group (Figure 10A). Evaluation of immune-related pathways showed that the levels of cytolytic, inflammation-promoting and T cell co-stimulatory activities were higher in the high-risk group, whereas the level of Type II interferon (IFN) response activity was lower in the low-risk group (Figure 10B). Univariate Cox analysis of the 359 DEGs with *p* < 0.05 identified 25 prognostic DEGs that were associated with cuproptosis and immunity. Analyses of correlations between these genes and immune cells and pathways involved in immune system function found that *IGKV1D-43*, *IGLV4-60*, *IGLV2-14*, *IGKV3-15,* and *IGHV3-30* were significantly associated with immunological status (Figure 10C).

## Discussion

With univariate Cox regression analyses, our study evaluated the predictive significance of cuproptosis-related genes based on the differential expression in tumors and adjacent nontumorous tissues of patients in TCGA. Univariate and lasso Cox regression analyses identified eight DEGs that were associated with prognosis in ccRCC patients. This led to the development of a five-gene risk signature for patients with ccRCC, with functional enrichment and immune correlation analyses indicating that this signature was strongly related to immune cells and immune system pathways. Expression levels of five signature genes were validated in vitor.

The present study also identified five genes associated with cuproptosis: *ATP7B*, *DBT*, *DLAT*, *LIAS*, and *PDHB*. *ATP7B* is a hepato-specific Golgi-localized ATPase involved in the regulation of copper homeostasis as well as in signaling processes (Polishchuk and Lutsenko, 2013). Increased resistance to platinum-based chemotherapy has been related to an increase in the expression of *ATP7B* in tumor cells (Nakayama et al., 2004). *DBT* is a subunit of transacylase (E2) (Matsuda et al., 1990)—a major enzyme involved in acid metabolism—and has been associated with maple syrup urine disease. *DLAT*, a mitochondrial protein involved in glucose metabolism, is overexpressed in gastric cancer cells and has been linked to the development, proliferation, and energy metabolism of gastric cancer cells (Goh et al., 2015). Moreover, the *PDH* complex, which includes *DBT* and *DLAT*, controls carbon entrance sites to the TCA cycle (Solmonson and DeBerardinis, 2018). *LIAS* expression has been associated with the activity of pyruvate dehydrogenase complex and pyruvate oxidation (Mayr et al., 2011). *PDHB* is a mitochondrial enzyme that catalyzes the conversion of glucose-derived pyruvate to acetyl-CoA and regulates the essential step of glycolysis in the citric acid cycle (Saunier et al., 2016). *PDHB* expression is lower in gastric cancer patients than in normal individuals, with lower *PDHB* expression being associated with poor patient prognosis (Goh et al., 2015). Despite extensive investigation of these five genes, their contribution to ccRCC remains unclear. *ATP7B*, *DLAT*, and *LIAS* were shown to be elevated, whereas *DBT* and *PDHB* were downregulated, in ccRCC samples. Moreover, the levels of expression of *DBT* and *DLAT* correlated with OS in ccRCC patients. In ccRCC and normal tissues, expression of *DBT*, *DLAT*, and *PDHB* were constrained by the KM curve for OS, which deserves additional investigation.

Cuproptosis is a newly described mechanism that mediates cell death and has been associated with mitochondrial metabolism (Tsvetkov et al., 2022). Cuproptosis may have important functions in microorganisms, as targets of copper-induced toxicity have been identified in various organisms, from bacteria to humans (Patteson et al., 2021). Antibacterial activity may therefore be liked to cuproptosis (Raffa et al., 2021). Genetic abnormalities in copper homeostasis, such as in patients with Wilson’s and Menke’s disease, can now be effectively treated by copper chelation (Aggarwal and Bhatt, 2018). However, the association between copper chelation and the treatment of cancer remains uncertain.

Little is currently known about the mechanisms of action of cuproptosis-related genes in ccRCC. The present study found that levels of expression of five cuproptosis-related genes, *ATP7B*, *DBT*, *DLAT*, *LIAS*, and *PDHB*, differed significantly in ccRCC and adjacent nontumorous kidney tissue and that a gene signature involving these five genes may be independently prognostic of OS in patients with ccRCC. Although the potential function of this gene signature was also assessed by immune activity and functional enrichment analyses, further research is needed to determine the precise mechanism of how these genes influence the incidence, development, treatment, and prognosis of ccRCC. Cuproptosis is closely associated with ccRCC, as many cuproptosis-related genes are differentially expressed in ccRCC and surrounding nontumorous tissues. Moreover, the present study found risk score based on this signature was closely related to the prognosis of ccRCC patients. Analysis of TCGA and ICGC showed that it could be an independent prognostic factor for predicting OS of ccRCC patients. Immunity is also tightly associated with these genes.

This study had several limitations. For example, publicly available data, such as RNA-seq and clinical data, may have certain drawbacks when analyzing the prognostic performance of gene signatures. Additional data, including primary data from patients with ccRCC, are needed to confirm the predictive significance of this gene signature. Moreover, additional research is required to determine the processes and activities by which cuproptosis-related genes promote tumor development.

This study identified a unique signature that can predict the prognosis of ccRCC patients, as well as five cuproptosis-related genes that may be associated with the mechanism of development of ccRCC. Moreover, these results provide a solid foundation for future research into the relationship between cuproptosis and immunity in ccRCC.

## Conclusion

Multiple bioinformatics methods were used to identify a cuproptosis-related signature that was associated with the prognosis of patients with ccRCC. This signature was found to be associated with incidence, survival, and immunity of ccRCC, suggesting that this signature may be an independent prognostic of OS in patients with ccRCC.

## Data Availability

Publicly available datasets were analyzed in this study. These data can be found at: RNA-seq and clinical data are available from TCGA (https://portal.gdc.cancer.gov/) and ICGC (https://dcc.icgc.org/releases/current/Projects/RECA-EU) datasets. Data on immune score can be acquired from ESTIMATE (https://bioinformatics.mdanderson.org/estimate). Codes in this research are available from corresponding author.

## References

[B1] Aggarwal A. Bhatt M. (2018). Advances in Treatment of Wilson Disease. Tremor Other Hyperkinet Mov. (N Y) 8, 525. 10.5334/Tohm.435 29520330 PMC5840318

[B2] Bondarczuk K. Piotrowska-Seget Z. (2013). Molecular Basis of Active Copper Resistance Mechanisms in Gram-Negative Bacteria. Cell Biol. Toxicol. 29, 397–405. 10.1007/s10565-013-9262-1 24072389 PMC3847284

[B3] Culotta V. C. Lin S.-J. Schmidt P. Klomp L. W. J. Casareno R. L. B. Gitlin J. (1999). Intracellular Pathways of Copper Trafficking in Yeast and Humans. Adv. Exp. Med. Biol. 448, 247–254. 10.1007/978-1-4615-4859-1_22 10079832

[B4] Dutcher J. P. (2013). Recent Developments in the Treatment of Renal Cell Carcinoma. Ther. Adv. Urology 5, 338–353. 10.1177/1756287213505672 24294292 PMC3825112

[B5] Friedman J. Hastie T. Tibshirani R. (2010). Regularization Paths for Generalized Linear Models via Coordinate Descent. J. Stat. Softw. 33, 1–22. 10.18637/jss.v033.i01 20808728 PMC2929880

[B6] Goh W. Q. Ow G. S. Kuznetsov V. A. Chong S. Lim Y. P. (2015). DLAT Subunit of the Pyruvate Dehydrogenase Complex Is Upregulated in Gastric Cancer-Implications in Cancer Therapy. Am. J. Transl. Res. 7, 1140–1151. 26279757 PMC4532746

[B7] Gulati S. Vaishampayan U. (2020). Current State of Systemic Therapies for Advanced Renal Cell Carcinoma. Curr. Oncol. Rep. 22, 26. 10.1007/s11912-020-0892-1 32048058

[B8] Hänzelmann S. Castelo R. Guinney J. (2013). GSVA: Gene Set Variation Analysis for Microarray and RNA-Seq Data. BMC Bioinforma. 14, 7. 10.1186/1471-2105-14-7 PMC3618321 23323831

[B9] Jensen L. J. Kuhn M. Stark M. Chaffron S. Creevey C. Muller J. (2009). STRING 8--a Global View on Proteins and Their Functional Interactions in 630 Organisms. Nucleic Acids Res. 37, D412–D416. 10.1093/nar/gkn760 18940858 PMC2686466

[B10] Lenth R. V. (2009). Response-Surface Methods inR, Usingrsm. J. Stat. Soft. 32, 1–17. 10.18637/jss.v032.i07

[B11] Ljungberg B. Campbell S. C. Cho H. Y. Jacqmin D. Lee J. E. Weikert S. (2011). The Epidemiology of Renal Cell Carcinoma. Eur. Urol. 60, 615–621. 10.1016/j.eururo.201110.1016/j.eururo.2011.06.049 21741761

[B12] Matsuda I. Nobukuni Y. Mitsubuchi H. Indo Y. Endo F. Asaka J. (1990). A T-To-A Substitution in the E1α Subunit Gene of the Branched-Chain α-ketoacid Dehydrogenase Complex in Two Cell Lines Derived from Menonite Maple Syrup Urine Disease Patients. Biochem. Biophysical Res. Commun. 172, 646–651. 10.1016/0006-291x(90)90723-z 2241958

[B13] Mayr J. A. Zimmermann F. A. Fauth C. Bergheim C. Meierhofer D. Radmayr D. (2011). Lipoic Acid Synthetase Deficiency Causes Neonatal-Onset Epilepsy, Defective Mitochondrial Energy Metabolism, and glycine Elevation. Am. J. Hum. Genet. 89, 792–797. 10.1016/j.ajhg.2011.11.011 22152680 PMC3234378

[B15] Nakayama K. Kanzaki A. Terada K. Mutoh M. Ogawa K. Sugiyama T. (2004). Prognostic Value of the Cu-Transporting ATPase in Ovarian Carcinoma Patients Receiving Cisplatin-Based Chemotherapy. Clin. Cancer Res. 10, 2804–2811. 10.1158/1078-0432.ccr-03-0454 15102688

[B16] Patteson J. B. Putz A. T. Tao L. Simke W. C. Bryant L. H. 3rd Britt R. D. (2021). Biosynthesis of Fluopsin C, a Copper-Containing Antibiotic from *Pseudomonas aeruginosa* . Science 374, 1005–1009. 10.1126/science.abj6749 34793213 PMC8939262

[B18] Polishchuk R. Lutsenko S. (2013). Golgi in Copper Homeostasis: a View from the Membrane Trafficking Field. Histochem. Cell Biol. 140, 285–295. 10.1007/s00418-013-1123-8 23846821 PMC3977151

[B19] Raffa N. Won T. H. Sukowaty A. Candor K. Cui C. Halder S. (2021). Dual-purpose Isocyanides Produced by Aspergillus fumigatus Contribute to Cellular Copper Sufficiency and Exhibit Antimicrobial Activity. Proc. Natl. Acad. Sci. U.S.A. 118, e2015224118. 10.1073/pnas.2015224118 33593906 PMC7923669

[B20] Ritchie M. E. Phipson B. Wu D. Hu Y. Law C. W. Shi W. (2015). Limma Powers Differential Expression Analyses for RNA-Sequencing and Microarray Studies. Nucleic Acids Res. 43, e47. 10.1093/nar/gkv007 25605792 PMC4402510

[B21] Saunier E. Benelli C. Bortoli S. (2016). The Pyruvate Dehydrogenase Complex in Cancer: An Old Metabolic Gatekeeper Regulated by New Pathways and Pharmacological Agents. Int. J. Cancer 138, 809–817. 10.1002/ijc.29564 25868605

[B22] Solmonson A. Deberardinis R. J. (2018). Lipoic Acid Metabolism and Mitochondrial Redox Regulation. J. Biol. Chem. 293, 7522–7530. 10.1074/jbc.TM117.000259 29191830 PMC5961061

[B23] Steinbrueck A. Sedgwick A. C. Brewster J. T. 2nd Yan K.-C. Shang Y. Knoll D. M. (2020). Transition Metal Chelators, Pro-chelators, and Ionophores as Small Molecule Cancer Chemotherapeutic Agents. Chem. Soc. Rev. 49, 3726–3747. 10.1039/c9cs00373h 32525153

[B24] Sung H. Ferlay J. Siegel R. L. Laversanne M. Soerjomataram I. Jemal A. (2021). Global Cancer Statistics 2020: GLOBOCAN Estimates of Incidence and Mortality Worldwide for 36 Cancers in 185 Countries. CA A Cancer J. Clin. 71, 209–249. 10.3322/caac.21660 33538338

[B25] Tanzi R. E. Petrukhin K. Chernov I. Pellequer J. L. Wasco W. Ross B. (1993). The Wilson Disease Gene Is a Copper Transporting ATPase with Homology to the Menkes Disease Gene. Nat. Genet. 5, 344–350. 10.1038/ng1293-344 8298641

[B26] Thul P. J. Lindskog C. (2018). The Human Protein Atlas: A Spatial Map of the Human Proteome. Protein Sci. 27, 233–244. 10.1002/pro.3307 28940711 PMC5734309

[B27] Tomczak K. Czerwińska P. Wiznerowicz M. (2015). Review the Cancer Genome Atlas (TCGA): an Immeasurable Source of Knowledge. wo 1A, 68–77. 10.5114/wo.2014.47136 PMC4322527 25691825

[B28] Tsvetkov P. Coy S. Petrova B. Dreishpoon M. Verma A. Abdusamad M. (2022). Copper Induces Cell Death by Targeting Lipoylated TCA Cycle Proteins. Science 375, 1254–1261. 10.1126/science.abf0529 35298263 PMC9273333

[B29] Tsvetkov P. Detappe A. Cai K. Keys H. R. Brune Z. Ying W. (2019). Mitochondrial Metabolism Promotes Adaptation to Proteotoxic Stress. Nat. Chem. Biol. 15, 681–689. 10.1038/s41589-019-0291-9 31133756 PMC8183600

[B30] Wang Q. Franz K. J. (2016). Stimulus-responsive Prochelators for Manipulating Cellular Metals. Acc. Chem. Res. 49, 2468–2477. 10.1021/acs.accounts.6b00380 27749047 PMC5482569

[B31] Wu T. Hu E. Xu S. Chen M. Guo P. Dai Z. (2021). clusterProfiler 4.0: A Universal Enrichment Tool for Interpreting Omics Data. Innovation 2, 100141. 10.1016/j.xinn.2021.100141 34557778 PMC8454663

[B32] Yoshihara K. Shahmoradgoli M. Martínez E. Vegesna R. Kim H. Torres-Garcia W. (2013). Inferring Tumour Purity and Stromal and Immune Cell Admixture from Expression Data. Nat. Commun. 4, 2612. 10.1038/ncomms3612 24113773 PMC3826632

[B33] Yruela I. (2009). Copper in Plants: Acquisition, Transport and Interactions. Funct. Plant Biol. 36, 409–430. 10.1071/FP08288 32688656

